# Society Meetings

**Published:** 1898-06

**Authors:** 


### SOCIETY MEETINGS.

THE Physicians' club held its annual meeting at the Hotel Ontario, Wednesday evening, May 18, 1898. The following officers were elected for the ensuing year: President, Dr. J. J. Walsh; vice-president, Dr. T. B. Carpenter; secretary, Dr. J. C. Clemesha. Council, Drs. J. H. Dowd, W. G. Ring, A. E. Diehl, T. F. Dwyer and C. E. Congdon.

Dr. Carpenter read a paper on Disinfectants, which was interestingly discussed. The retiring president, Dr. Diehl, then read his annual address, after which the meeting adjourned.

THE Buffalo Academy of Medicine held meetings during the month of May, 1898, as follows:

*Section on Surgery*—Meeting appointed for Tuesday evening, May 3d, was indefinitely postponed owing to the inability of the essayists to be present.

*Section on Medicine*—Tuesday evening, May 10th, program: Municipal hygiene, by Drs. H. R. Hopkins and Wm. G. Bissell. Annual meeting and election of officers.

*Section on Pathology*—Tuesday evening, May 17th, program: Diseases of the antrum of Highmore, by William C. Barrett, M. D., D. D. S. The pathology of the blood, by Dr. Albert E. Woehnert; Exhibition of specimens, by Dr. Edmond E. Blaau.

*Section on Obstetrics and Gynecology*—Tuesday evening, May 24th, program: Conservative treatment of the ovary, by Dr. H. E. Hayd. Subject to be announced, Dr. B. C. Johnson.

---

THE American Medical Temperance Association will hold a summer meeting at Prohibition Park, Staten Island, N. Y., July 5 and 6, 1898, under the presidency of Dr. N. S. Davis, of Chicago. Dr. T. B. Crothers, of Hartford, is the secretary, to whom all communications relating to the meeting should be addressed.

---

THE Cayuga County Medical Society held its ninety-second annual meeting at Auburn, Thursday, May 12, 1898. The following scientific program was observed: The president's address, Dr. John O. Palmer, Auburn, N. Y.; Multiple neuritis, its differential diagnosis and treatment, Dr. Edward D. Fisher, New York, N. Y.; Homing pigeons as medical messengers or bulletin bearers, illustrated by liberation of birds bearing messages, Dr. Charles L. Lang, Meridian, N. Y.; Report of a case, Dr. Frederick Sefton, Auburn, N. Y.

---

THE Medical society of the County of Erie will hold its regular semi-annual meeting at the room of the Buffalo academy of medicine Tuesday, June 14, 1898, under the presidency of Dr. Lucien Howe. The program of the meeting has not yet been issued.

---

THE American Medical Publishers' association will hold its fifth annual meeting at Denver, Col., on Monday, June 6, 1898 (the day preceding the meeting of the American medical association). Editors and publishers, as well as every one interested in medical journalism, cordially invited to attend and participate in the deliberations. Several very excellent papers are already assured, but more are desired. In order to secure a place on the program, contributors should send titles of their papers at once to the secretary, Charles Wood Fassett, St. Joseph, Mo.

---

THE fourth district branch of the New York State medical association held its fourteenth annual meeting at Buffalo, May 16, 1898. Dr. C. C. Frederick, the president, delivered his address, and Drs. Allen A. Jones, DeLancey Rochester and Charles G. Stockton, of Buffalo, read papers. Dr. William H. Thornton is the secretary.

---

THE National Confederation of State Medical Examining and Licensing boards will hold its eighth annual meeting at Denver, Col., June 6, 1898. The following program has been arranged by the secretary, Dr. A. Walter Suiter, Herkimer, N. Y.:

(1) Address of welcome, by William P. Munn, health commissioner of Denver.

(2) Response, by vice-president William Bailey, of Louisville.

(3) Report of committee on minimum standards of requirement, N. R. Coleman, chairman, Columbus, O.

(4) Discussion and action thereon.

(5) Report of secretary and treasurer.

(6) Annual address of the president, Uniformity the key to reciprocity, William Warren Potter, Buffalo.

(7) Results of the medical law of New Jersey, E. L. B. Godfrey, Camden.

(8) Results of the medical law of Massachusetts, E. B. Harvey, Boston.

(9) Results of the medical law of Kentucky, Joseph M. Mathews, Louisville.

(10) The Tennessee method, T. J. Happel, Trenton.

(11) Under what conditions is the state of Virginia likely to reciprocate as to standards with other states? R. S. Martin, Stuart.

(12) Some of the practical reasons why Latin and Greek should not be included in the standard of preliminary education required of medical students, Henry Beates, Jr., Philadelphia.

(13) Discussion thereon, J. A. Egan, Springfield, Ill.

(14) On the preparation of questions, Edward Cranch, Erie, Pa.

(15) Paper (subject to be announced), Charles K. Cole, Helena, Mont.

(16) Paper (subject to be announced), A. Walter Suiter, Herkimer, N. Y.

(17) Miscellaneous business.

(18) Election of officers.

(19) Adjournment.

The object of the confederation is to consider questions pertaining to state control in medicine and to compare methods in vogue in the several states; the collection and dissemination of information relating to medical education and to consider propositions that have for their purpose advancement of the standards in the United States. A cordial invitation is extended to all members and ex-members of state medical examining boards and to physicians, sanitarians and educators who are friendly to the objects named to attend the meeting and participate in its proceedings.

---

The American Medical Editors' association will hold its next annual meeting at Denver, Col., on Monday, June 6, 1898, the day preceding the opening session of the American medical association. The association will be entertained at a banquet on the evening of that day by the local committee on medical journals. Dr. Thomas H. Hawkins is the chairman of this committee.

---

The University of the state of New York will hold its thirty-sixth convocation in the senate chamber at Albany, Monday, Tuesday and Wednesday, June 27, 28 and 29, 1898. An excellent program has been issued and instructive discussions of the various subjects are expected.

---

The medical society of the County of Chemung held its annual meeting at Elmira, Tuesday, May 24, 1898. Dr. Frank W. Ross, president, read as his annual address a paper entitled Treatment of metritis. Dr. C. A. Murray, of Van Etten, read a paper on Chloroform in obstetrics.

